# Differential Proneness to Obesity in Two Rat Strains with Diverse Immune Responses

**DOI:** 10.3390/biology14050557

**Published:** 2025-05-16

**Authors:** Dina Tucović, Aleksandra Popov Aleksandrov, Dušanka Popović, Anastasija Malešević, Vesna Subota, Emilija Brdarić, Svetlana Soković Bajić, Milica Živković, Milena Kataranovski, Ivana Mirkov, Stanislava Stanojević, Jelena Kulaš

**Affiliations:** 1 Immunotoxicology Group, Department of Ecology, Institute for Biological Research “Siniša Stanković”—National Institute of the Republic of Serbia, University of Belgrade, 11000 Belgrade, Serbia; dina.mileusnic@ibiss.bg.ac.rs (D.T.); aleksandrap@ibiss.bg.ac.rs (A.P.A.); dusanka.popovic@ibiss.bg.ac.rs (D.P.); anastasija.malesevic@ibiss.bg.ac.rs (A.M.); m.kataranovski@gmail.com (M.K.); mirkovi@ibiss.bg.ac.rs (I.M.); stanislava.stanojevic@ibiss.bg.ac.rs (S.S.); 2 Institute for Medical Biochemistry, Military Medical Academy, 11000 Belgrade, Serbia; subota.vesna@gmail.com; 3 Group for Probiotics and Microbiota-Host Interaction, Department for Microbiology and Plant Biology, Institute of Molecular Genetics and Genetic Engineering, University of Belgrade, 11000 Belgrade, Serbia; emilija.brdaric@imgge.bg.ac.rs (E.B.); svetlana.sokovic@imgge.bg.ac.rs (S.S.B.); milica.zivkovic@imgge.bg.ac.rs (M.Ž.)

**Keywords:** Dark Agouti (DA) rat strain, Albino Oxford (AO) rat strain, obesity, metabolic syndrome, coagulation parameters, oxidative stress and inflammation, gut microbiota metabolic pathways

## Abstract

Although obesity and metabolic syndrome (comprising at least three of the following traits—abdominal obesity, elevated blood pressure, triglycerides and glucose/insulin resistance, and reduced high-density lipoprotein cholesterol in serum) are known to impact immune system activity, these conditions are often not considered when immune response characteristics are investigated in various rodent strains. In this work, metabolic syndrome indices are compared in 3 month-old (young) and 6 month-old (adult) rats of Dark Agouti (DA) and Albino Oxford (AO) strains, while parameters of coagulation, inflammation and oxidative stress were determined in young animals. Study reveals that both young and adult AO rats are obese, intolerant to glucose with higher levels of triglycerides and lower levels of high-density lipoprotein cholesterol when compared to age-matched DA rats. Parameters of coagulation, inflammation and oxidative stress that may contribute to the worsening of metabolic syndrome during aging are also higher in young AO rats. Metabolic syndrome observed in young and intensified in adult AO rats should be taken into consideration when analyzing alterations in immune reactivity during aging in this rat strain.

## Simple Summary

Rats of Dark Agouti (DA) and Albino Oxford (AO) rat strains significantly differ in the overall immune reactivity, but their metabolic profiling has never been performed. In this work, indices of metabolic syndrome (comprising at least three of the following traits—abdominal obesity, elevated blood pressure, triglycerides and glucose, and reduced “good” cholesterol in serum), are compared in 3 month-old (young) and 6 month-old (adult) DA and AO rats. Study reveals that young AO rats are obese, intolerant to glucose with higher levels of triglycerides and lower levels of “good” cholesterol when compared to DA rats of the same age, and all parameters were intensified in adult AO rats. It is therefore suggested that the next avenue of the research should examine the involvement of age-related metabolic changes in the immune alterations during aging in this rat strain.

## 1. Introduction

The high and increasing prevalence of metabolic syndrome [1] and obesity as its main component [2], depicts these conditions as worldwide health problems due to their association with type 2 diabetes mellitus, cardiovascular diseases, non-alcoholic fatty liver disease, kidney diseases and other chronic disorders [3,4]. Clinical diagnosis for metabolic syndrome is based on the expression at least three of the following traits—elevated waist circumference (as a measure of abdominal obesity), elevated triglycerides, and reduced levels of high-density lipoprotein cholesterol, elevated blood pressure, and elevated fasting glucose in serum/insulin resistance [5]. Considering that metabolic syndrome is caused by both genetic and environmental factors [6,7,8], various animal models have been developed to investigate the mechanisms that contribute to its etiology, pathogenesis, and possible treatment options. Some of the models use animals that spontaneously show features of metabolic syndrome or possess well-defined mutations in obesity-related genes, as well as the animals selectively bred for specific metabolic traits over several generations [6]. The other type of animal models includes diet-induced metabolic syndrome, such as high carbohydrate diet-, high fat diet-, or high carbohydrate/high fat diet-fed animals [7].

It has been acknowledged that, aside from metabolic alterations, animals fed with high-fat and/or high carbohydrate diet also display higher mortality rates following viral infections [9,10,11], have impaired antiviral response and response to vaccination [10,11,12], and exhibit delayed wound healing [13,14], which all suggest a significant impact of obesity and insulin resistance on the immune response. However, high-fat-fed rats [15] and mice [16] showed increased expression of inflammatory markers (i.e., pro-inflammatory cytokines) with the progression of obesity, whereas higher production of inflammatory cytokines by peripheral blood mononuclear cells was noted in obese humans when compared to normal weight controls [17]. It is noteworthy that metabolic syndrome and obesity-related metabolic diseases such as diabetes, are characterized by low-grade inflammation [8,9,18], pointing to the significance of the inflammatory cells in metabolic syndrome and obesity development, which is corroborated with human data on obesity-associated immune-related comorbidities [19]. Perturbations in the immune-metabolic cross-talk may lead to altered metabolic states and, consequently, to metabolic disorders [20]. This indicates the multiple bidirectional connections of immune and metabolic alterations in metabolic syndrome and put them in the “hen or egg”, or in *circulus vitious* situation.

Comparative studies of body weight, fat distribution and serum components relevant to obesity revealed substantial differences between several inbred, outbred and consomic rat strains [21,22] with no data concerning their immune reactivity. Conversely, a number of immune-related parameters have often been compared between different rat strains [23,24,25,26], but data regarding their metabolism, which might, possibly, affect the activity of the immune system [27] seems to be lacking.

Likewise, rats of two inbred major histocompatibility complex class II (MHC II) non-compatible rat strains, Dark Agouti (DA) and Albino Oxford (AO), exhibit differences in basal immune activity [28] and following immunization [29], but their comprehensive metabolic profiling has never been performed. The most notable difference in immune reactivity between these rat strains is high susceptibility of DA rats and complete resistance of AO rats to the induction of chronic inflammatory autoimmune-mediated arthritis and experimental autoimmune encephalomyelitis (EAE) [30,31]. Different behavioral patterns of these rat strains [30], higher capacity of the immune cells of DA rats to produce interleukin (IL)-17 and interferon γ (IFN-γ) [31,32] and the differences in the composition and the diversity of gut microbiota [33] were, to some extent, associated with the susceptibility and the resistance to the induction of autoimmune diseases in DA and AO rats, respectively. Lower capacity of AO rats’ cells to produce IL-17/ IFN-γ is also associated with lower intensity of contact hypersensitivity reaction to dinitrochlorobenzene [34]. Compared to DA rats, AO rats exhibit milder paw edema following Concanavalin A injection [35], milder skin response to oral intake of cadmium [36], and higher mortality following oral intake of warfarin [37]. Overall different immune reactivity is underlined by the fact that cells from AO rats, compared to cells from DA rats, respond to in vitro stimulation by mast cell mediators or zymosan [38], phorbol-myristate-acetate [39], indigenous microbiota [40], and probiotic bacteria [41] with augmented reactive oxygen species release. Therefore, it was tempting to explore if previously unrecognized putative differences in metabolic traits between these rat strains also exist. Our previous data corroborate this notion, as body mass of three-month old female DA rats varies between 170 g and 180 g, whereas the weight of age-matched female AO rats is in the range of 197–213 g [40]. Bearing in mind also the higher body mass of male AO when compared to age-matched male DA rats [42], which are lean and considered by some authors even obesity-resistant [43], the aim of this work was an examination of physiologic and metabolic parameters of rats of these two strains.

To this aim, basic parameters to define metabolic syndrome including obesity (body mass index and Lee obesity index), hyperglycemia/insulin resistance (the level of fasting blood glucose, glucose tolerance test, and insulin resistance test), and dyslipidemia (the levels of triglycerides, total cholesterol, high-density lipoprotein (HDL), and low-density lipoprotein (LDL) cholesterol) are analyzed. Given that the prevalence of metabolic syndrome slowly rises over the life span [44], we tested 3 month-old and 6 month-old DA and AO rats. Even though rats older than two months are considered sexually mature adults [45], at the age of 3 months they are actually at the high end of their adolescence period (rev in [46]), with still growing insulin-producing pancreatic β-cells [47], so we considered them as young adults, or simply young. Rats at the age of 6 months are fully developed and thereafter considered here as adults.

Inflammation and hypercoagulability are also important features of the metabolic syndrome that further predispose to atherothrombosis [48]. Roughly 30% of children and adolescents in the world are overweight and, owing to the incomplete development of antioxidant system in young, succumb to the chronic oxidative and inflammatory stress, which are responsible for the onset of all the complications typical of adult age, such as cardiovascular diseases [49]. As alteration present in childhood tends to perpetuate itself in adulthood [49], factors associated with metabolic syndrome and obesity, such as coagulation, inflammation and oxidative stress, are examined in young age, with an intention to explore if these are predisposing factors that may contribute to the worsening of metabolic syndrome symptoms during the transition to adulthood. Finally, given the involvement of gut microbiota in shaping the repertoire of diversity and function of immune cells [50], as well as its strong association with the metabolic traits of the organisms (even though its precise role in disease causation or as a merely secondary phenomenon is far from conclusive) [51], we have also performed analysis of gut microbial communities and their metabolic pathways in DA and AO rats.

## 2. Materials and Methods

### 2.1. Animals

Three-month old and six-month old rats of Albino Oxford (AO) and Dark Agouti (DA) rat strains were used in experiments. Having in mind sex differences in adipose tissue distribution and in metabolic inflammation [52], as well as the fact that sex steroids can alter both lipid and carbohydrate metabolism [53,54], only male rats were used in the study. As diet is among risk factors that strongly contribute to age-associated development of metabolic syndrome [55], both rat strains had free access to conventional rodent pellet (Veterinarski zavod, Subotica, Serbia) and water. Animals were housed conventionally (12-h light/dark cycle, ambient temperature of 22 ± 2 °C and 60% relative humidity) at the animal facility of the Institute for Biological Research “Sinisa Stankovic” (Belgrade, Serbia).

Animals were put in metabolic cages for 1 h daily for three consecutive days in order to acclimate to the apparatus. The following day, rats were left in metabolic cages where food and water intake, and feces and urine excretion were measured over 24 h.

All other procedures (except obtaining the blood from tail tip) were performed under inhalation anesthesia (Vetflurane, Virbac, Carros, France, minimal alveolar concentration of 3% isofluran).

### 2.2. Body Mass, Lee Obesity Index, and Abdominal and Thoracic Circumferences

Body mass was determined using a precise digital electronic scale with a capacity of 3 kg and a sensitivity of 0.01 g. Naso-anal body length, abdominal circumference (AC) (assessed on the rat abdomen in front of the hind legs), and thoracic circumference (TC, rat abdomen immediately behind the front legs) were measured using a non-extensible thread and readings were taken using a ruler with a sensitivity of 0.1 cm. Body mass index (BMI) was calculated by dividing body mass (expressed in g) by square length (expressed in cm^2^). Lee index, which assesses obesity in rats [56], was calculated using the following formula: ${Lee\;Index=}^{3\sqrt{\frac{weight\left(g\right)}{naso-anal\;length\left(cm\right)}}}$ × 1000.

### 2.3. Fasting Blood Glucose, Glucose Tolerance, and Insulin Resistance Test

Fasting blood glucose (following 4 h fast) was determined in blood obtained from the tail tip using a glucometer (Sensimac, IMACO GmbH, Lüdersdorf, Germany), immediately before the intraperitoneal injection of glucose (2 g/kg body weight) (Veterinarski zavod, Subotica, Serbia). Level of blood glucose was measured at 15, 30, 60, 90, and 120 min post-injection of glucose for assessing the glucose tolerance test (GTT). Following 4 days of recovery, rats were intraperitoneally injected with insulin (1 IU/kg body weight) (Sigma-Aldrich, St. Louis, MO, USA) and blood glucose level measured at 15, 30, 60, 90, and 120 min post-injection was used for determination of peripheral insulin sensitivity by insulin tolerance test (ITT). Area under the curve (AUC) for serum glucose and insulin was calculated by GraphPad Prism 8.0.2.

### 2.4. Clinical Biochemistry

Blood was withdrawn from the abdominal artery following a 4 h fasting for the whole blood determination of hematological parameters, and for plasma and serum preparation.

Hematological parameters were determined automatically by Siemens ADVIA 120 flow cytometer (Terytown, NY, USA) using commercially available reagents.

Total cholesterol, HDL cholesterol and triglycerides were measured in serum by Advia 1800, Clinical Chemistry Analyzer System (Siemens Healthcare Gmbh, Erlangen, Germany) using commercial tests. LDL cholesterol was determined using Friedwald formula [57]. Plasma fibrinogen was measured by Siemens-Dade Behring-BCT analyzer using Multifibren U test.

### 2.5. Coagulation Parameters

Bleeding time was determined by measuring the time from the moment of incision (the tail tip) to the moment bleeding stopped. Clotting time was defined as the time required for the blood to clot in the glass test tube. Prothrombin time (PT) and partial thromboplastin time (PTT) were determined in blood samples diluted in citrate buffer (1:5). PT was determined by a one-stage method using citrate plasma and Thromborel S reagents (Behring Diagnostics GmbH, Marburg, Germany) with Siemens equipment. The caolin-activated PTT was determined by a one-stage method using Pathrombin (Behring).

Measurement of anticoagulation factors antithrombin III and protein C was performed in citrate plasma by BCS XP System (Siemens Healthcare Gmbh, Germany) using commercially available reagents. Measurement of platelet adhesion and aggregation (aggregometry) was performed in whole blood diluted with citrate buffer following initiation of aggregation with adenosine-diphosphate on PFA-100 System (Siemens Healthcare Gmbh, Germany).

Parameters related to platelets are considered as in a review [58]. Namely, platelecrit measures total platelet mass as a percentage of volume occupied in the blood whereas platelet distribution width (PDW) is an indicator of heterogeneity in platelet size.

### 2.6. Oxidative Stress Measurement in Peripheral Blood

For determination of superoxide dismutase (SOD, EC 1.15.1.1) and catalase (CAT, EC 1.11.1.6) activity, heparinized blood was centrifuged at 400× *g* for 20 min and the remaining cell pellet was washed with cold physiological saline and erythrocytes were lysed by ultrapure water until the original volume was restored. SOD activity was determined by the adrenalin method [59]. One unit of activity was defined as the amount of enzyme necessary to decrease by 50% the rate of adrenalin autooxidation at pH 10.2. The activity of CAT was determined by the rate of H_2_O_2_ disappearance measured at 240 nm as described [60]. One unit of CAT activity is defined as the amount of enzyme that decomposes 1 mmol H_2_O_2_ per minute at 25 °C and pH 7.0.

Lipid peroxidation was evaluated by the thiobarbituric acid reaction as described [61]. Briefly, plasma samples were mixed with thiobarbituric acid-trichloroacetic acid (TBA-TCA) reagent and Tris-HCl (pH 7.4) and heated for 60 min at 95 °C. The absorbance of the supernatant obtained by centrifugation was measured at 535 nm using a spectrophotometer (Shimadtzu Corporation, Lakewood, CA, USA). Malondialdehyde (MDA) content was calculated by reference to a standard curve constructed by known amounts of MDA. Data were expressed as nmol of MDA/mg of protein. Protein concentration was determined by Lowry assay [62], using bovine serum albumin (Fraction V obtained from Sigma-Aldrich, St. Louis, MO, USA) as a reference.

### 2.7. Isolation and Culture of Peripheral Blood Mononuclear and Polymorphonuclear Cells

Peripheral blood mononuclear (PBMC) and polymorphonuclear cells (PMN) were isolated from the heparinized blood by dextran sedimentation and centrifugation (700× *g*, 20 min, 20 °C) on OptiPrep (Nycomed AS, Asker, Norway) density gradient. PBMC were harvested from the band at the interface of plasma and OptiPrep, while PMN were obtained from the pellet fraction following the lysis of erythrocytes with the isotonic NH_4_Cl solution. Cell purity, determined morphologically, was >95%. For cytokine production, cells (0.5 × 10^6^ cells/well in 96-well plates) were cultured 48 h in RPMI-1640 medium (supplemented with 2 mM glutamine, 20 µg/mL gentamycin and 5% (*v*/*v*) heat-inactivated fetal calf serum) solely or with 100 ng/mL of lipopolysaccharide (LPS).

### 2.8. Enzyme-Linked Immunosorbent Assay (ELISA)

Concentrations of cytokines IL-6 and tumor necrosis factor (TNF) were determined using commercially available ELISA sets (R&D Systems, Minneapolis, MN, USA) according to the manufacturer’s instructions. The standard curve generated using known amounts of the respective set provided recombinant cytokines was used to calculate cytokine titers. Cell cytokine production was determined in PBMC and PMN isolated from blood, and cytokine levels were determined in sera of DA and AO rat strains.

### 2.9. Bacterial Microbiota Analysis

Fresh feces were collected from ten DA and AO rats. Total genomic DNA extraction from fecal samples was performed with ZR Tissue DNA MiniPrep™ Kit (Zymo Research Corp., Irvine, CA, USA), according to the manufacturer’s instruction. The concentration of isolated DNA was measured on Qubit™ fluorometer (ThermoFisher/Invitrogen, Waltham, MA, USA). Samples were diluted to the concentration of 12 ng/μL in 20 μL of final volume and sent to Novogene Company (Cambridge, UK) for library preparation and 16S rRNA amplicon sequencing of the V3-V4 hypervariable region using 341 (forward, 5′–CCTAYGGGRBGCASCAG–3′) and 806 (reverse, 5′–GGACTACNNGGGTATCTAAT–3′) primers. Paired-end sequencing was performed on an Illumina NovaSeq 6000 platform, and the quality of sequencing was analyzed using the rarefaction method. Paired-end reads were merged using FLASH (V1.2.7) [63]. To obtain high-quality clean tags, quality filtering was performed [64], according to the QIIME [65]. For each representative sequence, QIIME Version 1.7.0 [66] in the Mothur method was performed against the SSUrRNA database of SILVA Database [67] for species annotation at each taxonomic rank. Alpha diversity calculations were done in QIIME and displayed with R software (Version 2.15.3), performed by Novogene Technology Co., Ltd. The raw 16S rRNA gene sequence data reported in this study have been deposited in the European Nucleotide Archive (https://www.ebi.ac.uk/ena) under study accession number PRJEB60516 (accessed on 10 March 2023).

Linear discriminant analysis effect size (LEfSe) was done using the Galaxy platform [68].

For the prediction of metabolic pathways, a phylogenetic investigation of communities by reconstruction of unobserved states (PICRUSt2) was performed [69]. Metabolic pathways were assigned based on the Kyoto Encyclopedia of Genes and Genomes (KEGG) Ortholog (KO) database. Read abundance data for all predicted pathways were converted to relative abundance (%) by the Galaxy server (https://mbac.gmu.edu/mbac_wp/) for LEfSe analysis.

### 2.10. Statistical Analysis

Results are expressed as mean ± standard deviation (S.D.) for ten animals per rat strain. Multiple comparisons between groups were done using one-way ANOVA followed by Tukey’s test, while comparison between two groups was performed using the Mann-Whitney U test (The Statistica 7.0, StatSoft Inc., Tulsa, OK, USA). A probability level less than 0.05 was considered significant.

## 3. Results

### 3.1. Body Mass and Lee Obesity Index in DA and AO Rats

Body mass is higher in AO compared to DA rats in both age groups (Table 1). As animals of both rat strains were provided *ad libitum* with the rodent chow (consisting of 3.2% fat, 49.3% carbohydrates, 20.3% proteins, and 12.9% fibers; energetic value 1405 KJ/100 g) and water, we sought to examine whether there are differences in consummatory behavior (food and water intake), or in the excretion of urine and feces that might result in higher body mass of AO rats. Although absolute values for food and water consumption (and urine volume in six-month-old animals) were higher in AO compared to DA rats, no differences could be noted when these values were normalized according to animals’ body weight (Table 1).

**Figure 1:**
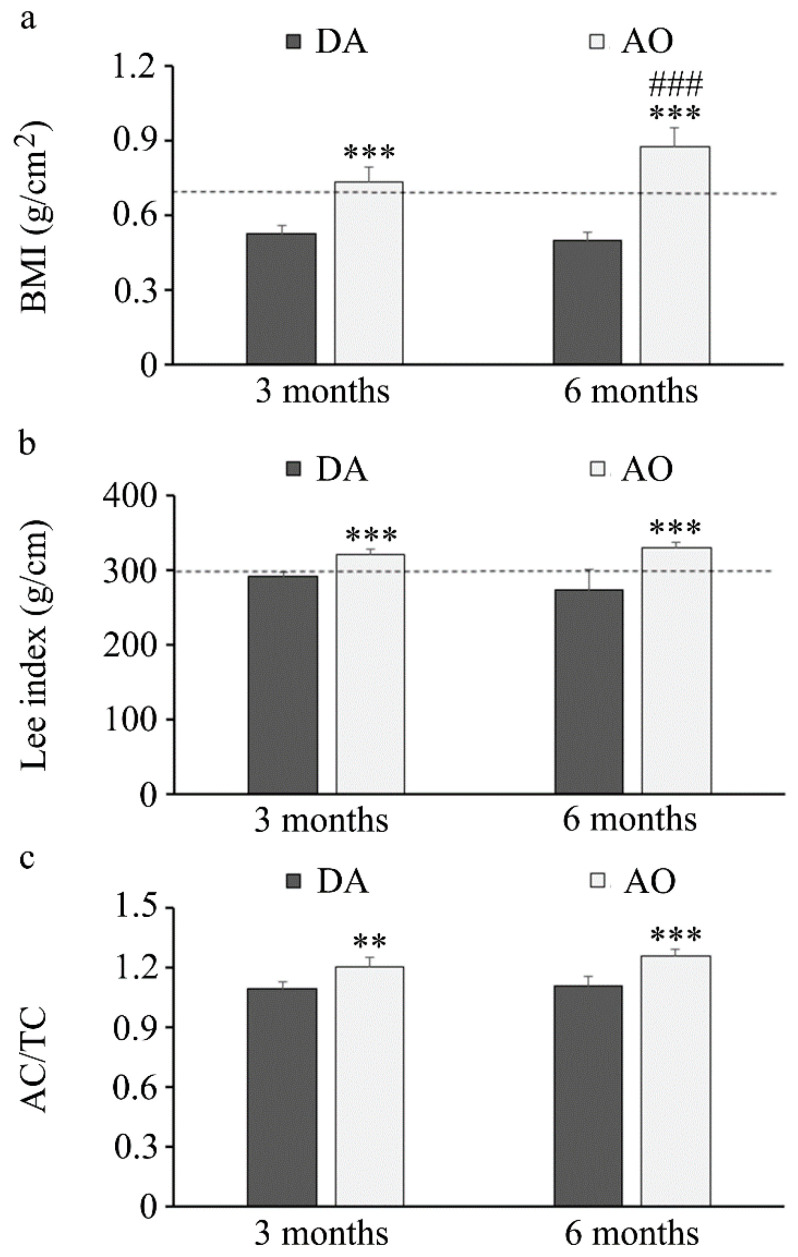
Obesity indices in three- and six-month-old DA and AO rats. (**a**) Body mass index. (**b**) Lee’s obesity index. (**c**) Abdominal/thoracic circumference. Results are presented as mean ± S.D. from ten animals per strain. The line represents a threshold value for obesity. Statistically significant at: ** *p* < 0.01 and *** *p* < 0.001 for AO vs. DA rats, and ^###^ *p* < 0.001 for 6-month-old vs. 3-month-old animals.

A comparison of body mass index and the Lee obesity index revealed higher values for both indexes in AO rats regardless of age (Figure 1a,b), with values in this strain over 0.68 (for BMI) and 300 (Lee index) that are defined as threshold values for obesity in rats [70,71]. Additionally, abdominal (AC) and thoracic (TC) circumferences were measured and their ratio was also higher in AO compared to DA rats (Figure 1c).

### 3.2. DA and AO Rats Differently Respond to Intraperitoneal Glucose and Insulin

**Figure 2:**
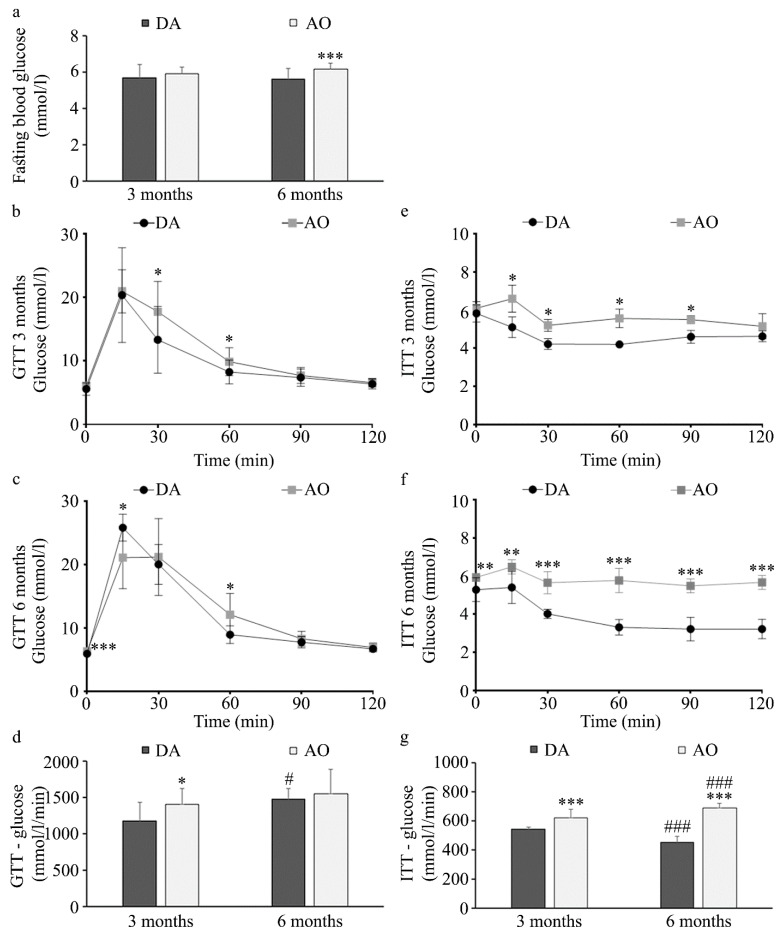
Response to glucose and insulin in DA and AO rats. (**a**) Fasting blood glucose. Glucose tolerance test in three- (**b**) and six-month-old animals (**c**,**d**) AUC for glucose tolerance test. Insulin tolerance test in three- (**e**) and six-month-old animals (**f**,**g**) AUC for insulin tolerance test. Results are presented as mean ± S.D. for 10 animals per strain. Statistically significant at: * *p* < 0.05, ** *p* < 0.01 and *** *p* < 0.001 for AO vs. DA rats, and ^#^ *p* < 0.05 and ^###^ *p* < 0.001 for 6-month-old vs. 3-month-old animals.

Parameters relevant to diabetes including fasting glucose, glucose tolerance, and insulin resistance in these two strains were examined next. No differences were found in fasting blood glucose levels in three-month-old animals, but significantly higher blood glucose was noted in six-month-old AO than in DA rats (Figure 2a). Differences between rat strains in response to glucose were noted at both three-month (Figure 2b) and six-month (Figure 2c) -old animals. In both rat strains at three months of age, a similar increase in blood glucose level was noted 15 min after intraperitoneal injection of 2 g/kg b.w. glucose (Figure 2b), but in six-month-old animals this increase was higher in DA compared to AO rats (Figure 2c). Initial increase was followed by a more pronounced decrease in DA rats (at 30 and 60 min in three-month-old animals, and at 60 min in six-month-old rats). This dynamic resulted in higher area under the curve (AUC) values for glucose in three-month-old AO compared to DA rats (Figure 2d), but not in six-month-old animals in which AUC for the glucose tolerance test was similar for both strains (Figure 2d). This was probably a result of the more pronounced glucose increase at 15 min in adult DA animals, while unchanged in AO rats. Nevertheless, a more pronounced decrease in blood glucose following intraperitoneal insulin injection was noted in DA rats regardless of age (Figure 2e,f) resulting in larger AUC for insulin in AO rats than in DA (Figure 2g). With aging, AUC for insulin decreased in DA rats, in contrast to AO rats in which higher values were noted in six-month-old compared to three-month-old animals.

### 3.3. Serum Lipid Levels in DA and AO Rats

**Figure 3:**
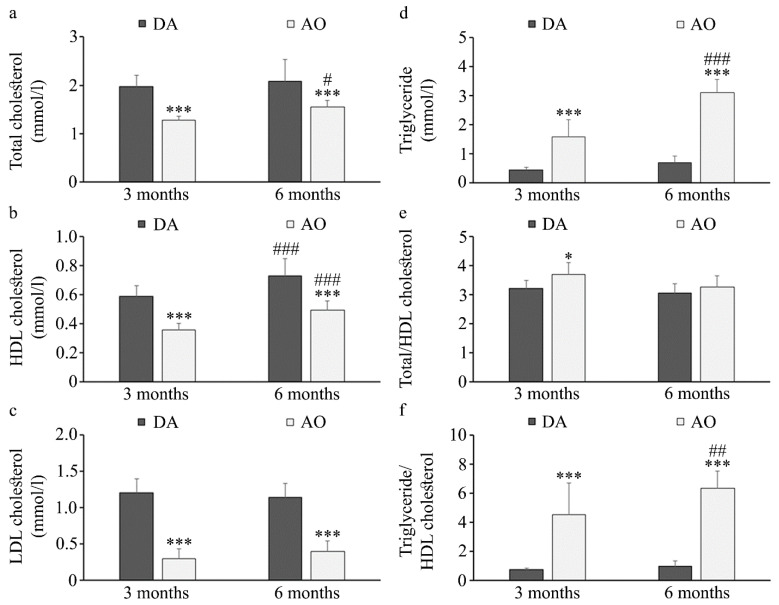
Serum lipid levels in DA and AO rats. (**a**) Total cholesterol, (**b**) HDL cholesterol, (**c**) LDL cholesterol, (**d**) Triglycerides, (**e**,**f**) Atherogenic indexes. Results are presented as mean ± S.D. for 10 animals per strain. Statistically significant at: * *p* < 0.05, and *** *p* < 0.001 for AO vs. DA rats, and ^#^ *p* < 0.05, ^##^ *p* < 0.01 and ^###^ *p* < 0.001 for 6-month-old vs. 3-month-old animals.

Having in mind that dyslipidemia is another hallmark of metabolic syndrome [72], next we measured levels of triglycerides, total cholesterol, high-density lipoprotein (HDL), and low-density lipoprotein (LDL) cholesterol. Obtained results showed lower levels of total cholesterol (Figure 3a), HDL cholesterol (Figure 3b) and LDL cholesterol (Figure 3c) but higher triglycerides (Figure 3d) in AO in comparison to DA rats regardless of age. In AO rats the levels of all lipids (except LDL cholesterol) increased with aging, while in DA rats only the level of HDL cholesterol was higher in six-month-old when compared to three-month-old animals. When atherogenic indexes were calculated (ratio of total and HDL cholesterol, and triglyceride and HDL cholesterol) higher values were obtained in AO rats (Figure 3e,f). It must be taken into account that even though levels of both triglycerides and HDL cholesterol increased with aging in AO rats, more substantial increase of triglycerides than HDL cholesterol resulted in increase in their relative ratio in 6-month-old rats.

### 3.4. Hematological Parameters in DA and AO Rats

Analysis of hematological parameters revealed a lower number of total leukocytes in the peripheral blood of AO rats (Table 2). A relative number of neutrophils and eosinophils was higher, but number of lymphocytes was lower in AO when compared to DA rats. These differences resulted in a higher neutrophil-to-lymphocyte ratio in AO than in DA rats. Additionally, the calculation of the systemic immune-inflammation index (neutrophils × platelet/lymphocytes) revealed higher values in AO compared to DA rats.

The lower number of red blood cells was noted in AO rats (Table 2) accompanied by higher hemoglobin, mean corpuscular volume (MCV), mean corpuscular hemoglobin (MCH) and mean corpuscular hemoglobin concentration (MCHC).

### 3.5. DA and AO Rats Differ in Hemostasis

**Figure 4:**
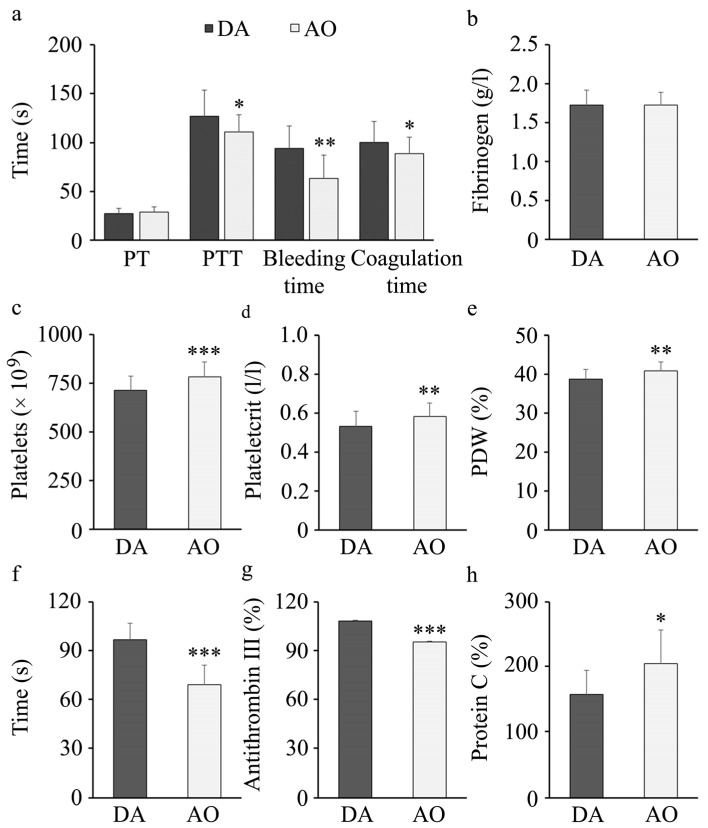
Hemostasis parameters in DA and AO rats. (**a**) Prothrombin (PT), partial thromboplastin (PTT), bleeding and clotting time. (**b**) Fibrinogen levels in plasma. (**c**) Platelet counts in peripheral blood, (**d**) Plateletcrit (PCT) and (**e**) Platelet distribution width (PDW). (**f**) ADP-stimulated aggregation of platelets. (**g**) Antithrombin III and (**h**) Protein C in plasma. Results are presented as mean ± S.D. for 10 animals per strain. Statistically significant at: * *p* < 0.05, ** *p* < 0.01 and *** *p* < 0.001 for AO vs. DA rats.

Impaired hemostasis is seen in individuals with metabolic syndrome [73]. To examine if there are differences in hemostasis in DA and AO rats, we first measured prothrombin (PT), partial thromboplastin (PTT), bleeding, and clotting time (Figure 4a). No differences were noted in PT values obtained in DA or AO rats. Still, lower PTT, bleeding and clotting times were observed in AO rats. Further examinations revealed no differences in fibrinogen levels between these strains (Figure 4b). Examination of platelet numbers and indices (plateletcrit and platelet distribution width/PDW) revealed higher values in AO compared to DA rats (Figure 4c–e). To examine differences in platelet activation status, we stimulated them with adenosine diphosphate (ADP) and measured the time until aggregate formation. Faster aggregation of platelets following agonist stimulation was noted in AO compared to DA rats (Figure 4f). In addition to higher platelet activation, lower antithrombin III, an anticoagulation factor, were noted in AO than in DA rats (Figure 4g), whereas another anticoagulant, protein C was higher in AO rats (Figure 4h).

### 3.6. Gut Microbiota in DA and AO Rats

Gut bacterial microbiota has been linked with metabolic syndrome and obesity [74] and its composition is different in healthy DA and AO rats [75]. More detailed analysis of fecal samples revealed no differences in alpha-diversity indices (number of observed species, Shannon and Chao1 indexes) between strains (Table 3). At the phylum level, a similar relative abundance of Firmicutes, Bacteroidetes, and Proteobacteria between rat strains was noted, while Actinobacteria were lower in AO rats (Table 3).

**Figure 5:**
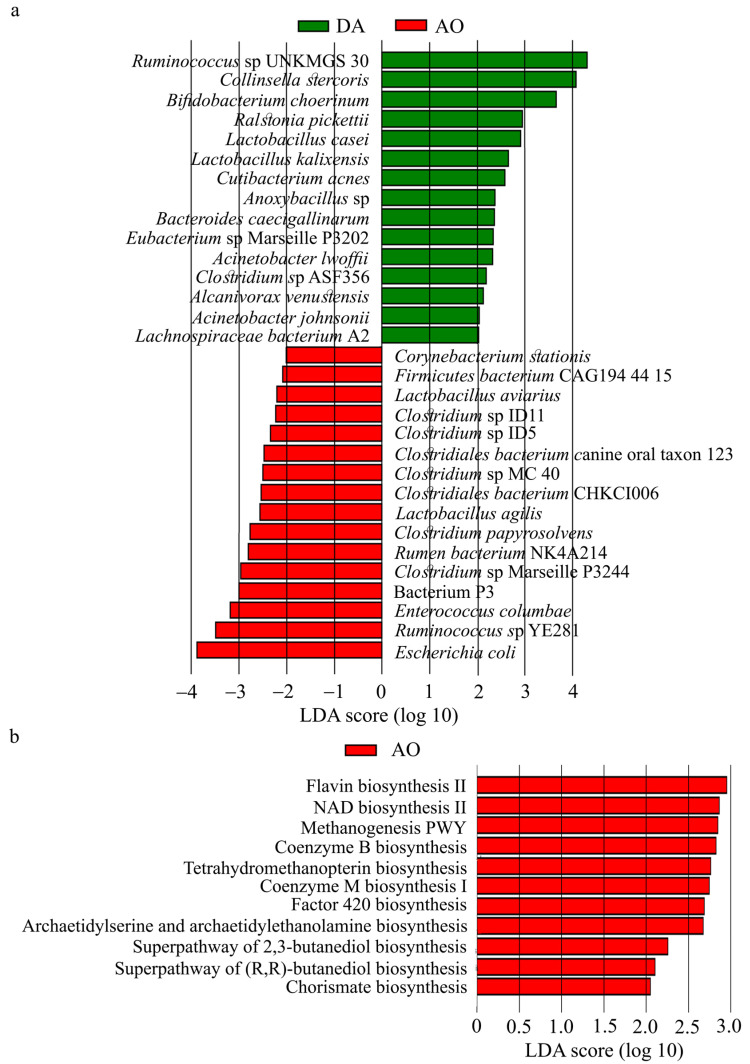
Bacterial species (**a**) and metabolic pathways (**b**) that differ between DA and AO rats.

Using LEfSe analysis, species that are differently abundant in DA and AO rats were identified (Figure 5a). Abundance of a total of 31 species was significantly different between DA (higher abundance of 15 species) and AO (higher abundance of 16 species) rats. Of 15 species identified in DA rats, 7 (47.6%) belongs to phylum Firmicutes (3 species from the class Bacilli and 4 belonging to class Clostridia), 1 (6.7%) to phylum Bacteroides (class Bacteroidia), 4 (16.7%) to Proteobacteria (class Gammaproteobacteria) and 3 (20.0%) to Actinobacteria (2 species belong to class unidentified Actinobacteria, and 1 from class Coriobacteriia). In AO rats, from 16 differently abundant species 1 (6.2%) belongs to each phylum, Bacteroidetes (class Bacteroidia), Actinobacteria (unidentified Actinobacteria), and Proteobacteria (class Gammaproteobacteria). The remaining species (81.2%) belongs to phylum Firmicutes: class Clostridia (9 species), Bacilli (3 species), and Erysipelotrichia (1 species). Prediction of metabolic pathways (using PICRUST) in these two strains indicated 11 pathways that might be more active in AO rats (Figure 5b). It is interesting to note that 5 of 11 (45.4%) pathways are included in methanogenesis.

### 3.7. Oxidative Stress and Inflammation in Peripheral Blood

Metabolic syndrome and diabetes are characterized by low-grade systemic inflammation and oxidative stress [8].

**Figure 6:**
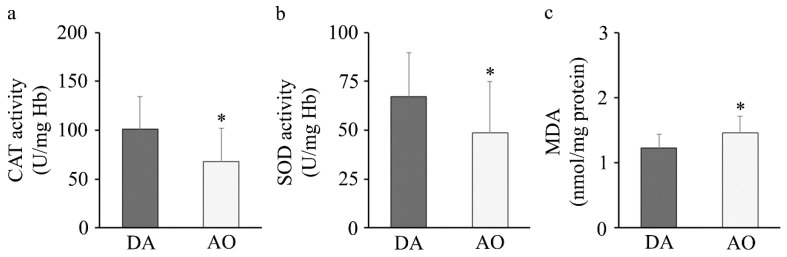
Oxidative stress. (**a**) Catalase (CAT) and (**b**) superoxide dismutase (SOD) activity in erythrocytes and (**c**) malondialdehyde (MDA) in plasma. Results are presented as mean ± S.D. for 10 animals per strain. Statistically significant at: * *p* < 0.05 for AO vs. DA rats.

Determination of oxidative stress parameters revealed a lower activity of erythrocyte catalase and superoxide dismutase and higher MDA levels in the plasma of AO rats (Figure 6).

**Figure 7:**
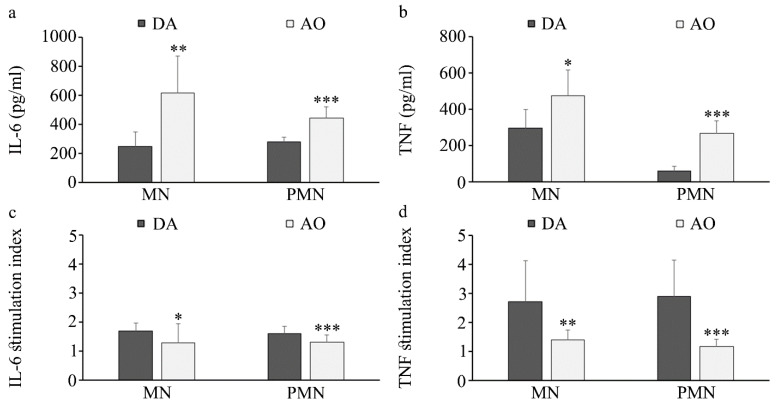
Cytokine production by peripheral blood mononuclear (PBMC) and polymorphonuclear (PMN) cells. (**a**) IL-6 and (**b**) TNF production, and responsiveness to LPS (**c**,**d**) stimulation. Results are presented as mean ± S.D. for 10 animals per strain. Statistically significant at: * *p* < 0.05, ** *p* < 0.01 and *** *p* < 0.001 for AO vs. DA rats.

No differences in IL-6 and TNF levels in plasma were noted between DA (34.4 ± 6.4 pg/mL for TNF and 18.6 ± 3.4 pg/mL for IL-6) and AO rats (30.6 ± 8.0 pg/mL for TNF and 18.5 ± 7.0 pg/mL for IL-6, respectively). However, when cytokine production by peripheral blood mononuclear and polymorphonuclear cells was examined, higher basal IL-6 and TNF production was detected in cells isolated from AO rats (Figure 7a,b). Leukocyte responsiveness to stimulation with inflammatory stimulus, lipopolysaccharide/LPS, was lower in AO rat strain (Figure 7c,d).

## 4. Discussion

Our study revealed that, under *ad libitum* feeding conditions, AO rats are obese, exhibit impaired glucose tolerance and reduced insulin sensitivity, whereas systemic triglyceride levels are higher and the levels of total cholesterol, HDL and LDL cholesterol are lower, when compared to DA rats. Based on body mass index, DA rats might be considered to have normal weight throughout the life, while both young and adult AO rats can be considered overweight as BMI in these animals exceeds 0.68 g/cm^2^ that is defined as the threshold for obesity [70,76]. Of note, our unpublished observations confirm that differences in body mass index exist even in younger animals as well (0.34 ± 0.03 vs. 0.44 ± 0.02, *p* = 0.0002, for one-month-old, and 0.46 ± 0.03 vs. 0.63 ± 0.03, *p* = 0.0001, for two-month-old DA and AO rats, respectively). In accordance with previous data that BMI in rats increases in the first 90 days of life and thereafter remains relatively constant [76], body mass of DA rats increased for only 21% in a period spanning from 3 to 6 months of age and BMI remained unchanged. In contrast to that, body mass of AO rats increased for 51% in the same period and a continual increase in BMI was observed. Lee’s obesity index and abdominal/thoracic circumference ratio were also higher in both young and adult AO compared to age-matched DA rats. Aside from being additional measure of obesity, Lee’s index has a positive correlation with retroperitoneal fat [77], and also confirms that even younger AO rats can be considered overweight (323.3 ± 5.6 for one-month-old and 314.1 ± 6.1 for two-month-old AO rats, unpublished observations). Judging by all morphometric parameters, young AO rats are obese, and obesity is exaggerated toward the adulthood. Considering that both rat strains are offered the same laboratory animal chow, the obesity of AO rats is not a result of a specific diet.

In line with previous results showing that the levels of fasting blood glucose were not different between young control and high-fat diet-fed rats [74,78], the levels of fasting blood glucose were similar in young DA and AO rats, whereas aging, i.e., the transition to the adulthood, increased it solely in AO rats. Age-dependent increase in glucose level and decrease in the level of insulin was noted in WBN/Kob rats, a strain that spontaneously develops type 2 diabetes mellitus at around six months of age [79]. Despite similar fasting blood glucose, a greater area under the curve in glucose and insulin tolerance tests suggests that AO rats are glucose intolerant and express insulin resistance, which is in concordance with data obtained from animals with diet-induced metabolic syndrome [8,74,78,80,81]. Abdominal obesity is highly correlated with insulin resistance [5] and in all obesity/metabolic syndrome models, the levels of triglycerides are elevated [8,81,82], while alteration in other lipids depends on the model used. Whereas in young and adult AO rats, higher levels of triglycerides were accompanied by lower total levels of cholesterol, HDL and LDL cholesterol when compared to age-matched DA rat strain, in cafeteria diet-fed Wistar rats the increase was noted in total cholesterol, and in HDL and LDL levels [8], while in Sprague Dawley rats on a high-fat diet, higher total cholesterol was accompanied by lower levels of HDL and unchanged levels of LDL [82]. Increased levels of total cholesterol and LDL, but lower levels of HDL were noted in Sprague Dawley rats on high-sucrose and high-fat diets [81]. As DA and AO rats were maintained on the same standard laboratory chow with similar caloric intake/food consumption, their differences in body mass are probably a result of different feeding efficiency, whereas differences in lipid profile are a consequence of their different metabolism. It is interesting to note that in DA rats, with normal weight, no alterations could be noted in lipid levels with aging, whereas in AO rat strain, in which BMI is over the threshold for obesity, an increase in BMI with aging is accompanied by an increase in lipid levels, suggesting worsening of the symptoms related to metabolic syndrome. This finding is in line with data showing that characteristics of metabolic syndrome have a linear relationship with aging [83]. An increase in serum lipid levels with aging was also noted in male WBN/Kob rats that spontaneously develop diabetes [79].

Complex connections between lipid status, oxidative stress and coagulation lead to further aggravation of metabolic syndrome toward atherosclerosis, diabetes and cardiovascular diseases. Interrelation between inflammation and metabolic abnormalities can be a causative factor for vascular damage, and one indicator of these effects might be endothelial dysfunction along with a pro-coagulant state [84]. Hence, we next wanted to examine if AO rat express altered parameters of coagulation and inflammation in young age as predisposing factors that may contribute to the worsening of metabolic syndrome symptoms during their transition toward the adulthood.

Indeed, lower PTT, shorter bleeding and clotting times, higher platelet activity and altered levels of anticoagulant factors were noted in AO rats compared to DA rat strain, indicating hypercoagulability and pro-thrombotic state. Both PT and PTT are in vitro tests that measure coagulation cascade (a time to generate fibrin) associated with quantities of coagulation factors, prothrombin and fibrinogen, but only PT measures extrinsic and intrinsic coagulation pathways [85]. As fibrinogen concentration is not different between DA and AO strains, putative differences in the concentration of other factors must have contributed to lower PTT in AO rats. Bleeding time depends on the number and the activity of platelets [85], whereas ADP-stimulated platelet aggregation time indicates their functional activity. Higher platelet distribution width (PDW) in AO rats, indicating the range of the platelet size, points to their increased activation, destruction or consumption, [86], which, together with greater platelet number and mass (plateletocrit) leads to more efficient platelet aggregation, faster coagulation and shorter bleeding time in AO compared to DA rats.

In line with that, the levels of coagulation inhibitor antithrombin III was lower in AO rat strain and it was paralleled with higher values of another coagulation inhibitor, protein C, as previously observed in obese humans [87,88].

Changes in lipid profile along with assumed prothrombotic state might predispose obese AO rats to cardiovascular diseases, in broad agreement with data on comorbidities in patients affected by metabolic syndrome [73]. Having in mind that oxidative stress is known to damage erythrocyte membranes and cause cell aging, especially when associated with increased BMI [89], it can be assumed that lower activity of superoxide dismutase and catalase noted in erythrocyte membranes of AO rats may be responsible for lower number of red blood cells in AO when compared to DA rats. In support of the assumption of cell damage in peripheral blood, higher levels of lactate dehydrogenase were noted in plasma of AO rats (644.3 ± 258.9 U/L) than in DA rats (440.9 ± 84.7 U/L, *p* = 0.0189). Likewise, higher values of hemoglobin, and other indices of hemoglobin content in erythrocytes (MCV, MCH and MCHC) noted in AO rats might be adaptation to slightly lower number of red blood cells, in order to provide enough oxygen for tissues. It should be noted, that even though higher than in DA rats, all values are within the normal range for laboratory rats [90].

Obesity seems to be one of the parameters that might impact immune reactivity [9,10,11,12,13,14]. However, comparable levels of plasma TNF and IL-6 contradicted the expectation of low-grade inflammation in AO rats, at least judged by cytokine content, which is one of the hallmarks of metabolic syndrome [8,81]. Similar levels of plasma TNF and even lower levels of IL-6, when compared to DA rats, were previously noted in naïve female AO rats [29], which was in contrast to data obtained in obese/metabolic syndrome models in which higher IL-6 and TNF were detected in the blood of obese animals [8,9,81]. Differences might be a consequence of a diet, as standard rodent chow was used in the present study, whereas food enriched in carbohydrates and/or fat was used for inducing obesity in the previous ones [8,9]. Consumption of high carbohydrates/fat diet was shown to cause damage of intestinal epithelium, resulting in increased levels of LPS, IL-6 and TNF in plasma [81]. It must be also taken into account that systemic inflammation is not necessarily a measure of tissue inflammation, and in this regard, higher IL-6 expression was noted in the liver of AO than in DA rats [42]. In line with data obtained by obese humans [17], results of the present study revealed higher basal IL-6 and TNF production by peripheral blood mononuclear (PBMC) and polymorphonuclear cells (PMN) from AO rats, and their lower responsiveness to LPS. This may indicate tolerance to LPS stimulation, as suggested for PMN from high-fat diet-induced obese rats, and was probably result of alteration in toll-like receptor (TLR) 4 (i.e., LPS receptors) pathways [91]. It is worth noting that our previous results, similar to those from the present study, revealed that unstimulated resident peritoneal cavity cells of AO rats produced higher levels of TNF and comparable levels of IL-6 when compared to rats of DA strain [40,92], but failed to respond to LPS stimulation to the extent of the response of DA rat cells [92]. The latter was also associated with alterations in TLR4 pathways, as AO rats have lower proportion of resident peritoneal cavity cells that express TLR4 when compared to DA rats [93]. Nevertheless, a relative neutrophilia (lower number of total leucocytes but the higher proportion of neutrophils in blood), higher neutrophil-to-lymphocyte ratio and higher systemic immune-inflammation index speaks in favor of low level systemic inflammation in AO rats, in spite of lack of differences in systemic cytokine levels between these strains. High values of these indices are observed in various inflammatory conditions [94,95,96]. In addition, lower activity of anti-oxidative enzymes in erythrocytes which probably results in cell damage, as indicated by higher LDH levels in AO rats’ plasma, accompanied by higher plasma MDA levels in these rats, supports the association of obesity with oxidative stress [8] also in AO strain.

Previous analysis of gut microbiota showed different microbial compositions and diversity in DA and AO rats [75]. As gut microbiota differs between obese and normal-weight humans [97] and animals [74,80], we have analyzed microbial communities in the gut of these rat strains, especially in context of analysis of their metabolic pathways that might contribute to obesity/metabolic syndrome. Results indicated a higher abundance of the representatives from the class Clostridia and Erisipelotrichia in AO rats, which is in accordance with the domination of these bacteria in animals on high-fat diet [74]. Bacterial phylum less abundant in AO rat, Actinobacteria, was reported to be as lean-associated [98]. Additionally, the prediction of metabolic pathways suggested that there are pathways more active in AO rats, including those relevant to methanogenesis. Enrichment in genes included in methane metabolism was noted in obese children (when compared with normal-weight subjects) [98], and enriched fecal methanogen bacteria were noted in animals on a high-fat diet as well [99]. Higher production of methane (measured as a higher concentration of gas in breath) was associated with higher BMI in humans [100].

Taken together, according to well accepted criteria—abdominal obesity, insulin resistance, elevated triglycerides and reduced high-density lipoprotein cholesterol, AO rats may be qualified as the strain that express symptoms of metabolic syndrome as young. This was not only further substantiated by other factors relevant to obesity and metabolic syndrome (altered hemostasis, increased oxidative stress, low level systemic inflammation and enrichment in gut microbiota metabolic pathways relevant to methanogenesis), but probably also aggravated by these factors during the transition into the adulthood, as elevated fasting glucose in serum was seen only in adults. It must be taken into account that metabolic syndrome and obesity predispose to the development of autoimmunity [101,102], whereas AO rats are resistant to commonly induced autoimmune diseases, such as adjuvant or collagen-induced arthritis, EAE, or low doses streptozotocin-induced autoimmune diabetes [30,103,104]. Hence, metabolic traits of AO rats make them prone for development of type 2 diabetes and, possibly, other conditions associated with metabolic syndrome, while factors most likely independent from their metabolic characteristics contribute to the resistance to the induction of autoimmune response.

Extensive research revealed diverse characteristics of AO rats which, in contrast to characteristics of DA rats, actually contribute to the resistance to the induction of autoimmune diseases, and are described both in periphery (for example, low IL-2, IFN-γ, TNF, IL-17, inability to expand Th1 response, low proportion of CX3CR1^+^ NK cells in draining lymph nodes) [104,105], as well as in target organs (i.e., higher expression of chemokine CXCL12 and impaired expression of chemokine ligand CCL2 in spinal cord tissue, low expression of heat-shock protein 47 in joint tissue) [106,107,108]. This is probably a result of the differences in the expression of both MHC and non-MHC genes between DA and AO rats. Nevertheless, it has been previously described that aging may overcome genetic resistance to EAE in this strain, as immunization of 24–26 month-old AO rats lead to mild chronic disease [109]. Having in mind the complexity of the biological milieu that dictates susceptibility/resistance to development of autoimmune diseases, it would be challenging to explore if metabolic syndrome observed in young and intensified in adult AO rats, in spite of not being directly involved in the skewing toward autoimmunity in young, may to some extent contribute to the changes in the autoimmune disease susceptibility of these rats observed in old age.

## 5. Conclusions

To the best of our knowledge, this is the first study showing that DA and AO rat strains differ not only in immune response (as extensively published previously) but in metabolism as well. Obesity, insulin resistance and dyslipidemia along with changes in associated parameters indicate AO rats as a rat strain with metabolic syndrome. Metabolic syndrome observed in young and intensified in adult AO rats should be taken into consideration when analyzing aging-induced alterations in immune reactivity in this rat strain.

## Figures and Tables

**Table 1:** Morphometric parameters and basal metabolism in DA and AO rats ^1^.

	DA		AO	
	Absolute	Per 100 g b.w.	Absolute	Per 100 g b.w.
3-month old animals				
Body mass (g)	196.9 ± 20.1		245.7 ± 24.2 *	
Naso-anal length (cm)	21.2 ± 0.6		21.9 ± 0.9	
Food intake (g/day)	17.3 ± 1.8	8.9 ± 1.7	22.6 ± 5.5 *	9.7 ± 2.1
Water intake (mL/day)	23.8 ± 7.4	12.3 ± 4.7	33.1 ± 8.0 *	14.4 ± 4.1
Urine volume (mL/day)	5.5 ± 2.1	2.8 ± 1.2	6.7 ± 1.1	3.0 ± 1.0
Feces (g/day)	9.2 ± 2.6	4.6 ± 1.4	10.1 ± 3.5	4.4 ± 1.4
6-month old animals				
Body mass (g)	235.4 ± 7.2		371.5 ± 35.2 ***	
Naso-anal length (cm)	22.3 ± 0.4		24.4 ± 0.6 ***	
Food intake (g/day)	17.5 ± 3.7	7.5 ± 1.7	25.9 ± 4.5 ***	7.3 ± 1.4
Water intake (mL/day)	21.1 ± 3.9	9.0 ± 1.8	31.5 ± 10.0 *	8.9 ± 3.0
Urine volume (mL/day)	4.5 ± 2.4	1.9 ± 1.0	8.3 ± 4.1 *	3.0 ± 1.0
Feces (g/day)	9.7 ± 3.0	4.1 ± 1.3	13.5 ± 4.1	3.8 ± 1.1

^1^ Results are presented as mean ± S.D. for 10 animals per strain. Statistically significant at: * *p* < 0.05 and *** *p* < 0.001 for AO vs. DA rats.

**Table 2:** WBC and RBC in healthy DA and AO rats ^1^.

	DA	AO
White blood cells (WBC)		
Total WBC (×10^9^/L)	6.7 ± 1.6	4.2 ± 1.1 ***
Neutrophils (%)	37.7 ± 6.6	44.3 ± 7.4 *
Lymphocytes (%)	60.1 ± 6.6	52.4 ± 7.9 *
Monocytes (%)	0.8 ± 0.2	1.0 ± 0.3
Eosinophils (%)	1.1 ± 0.3	1.8 ± 0.4 ***
Basophils (%)	0.3 ± 0.1	0.3 ± 0.1
Red blood cells (RBC)		
RBC number (×10^12^/L)	8.1 ± 0.4	7.9 ± 0.5 *
Hemoglobin (g/L)	136.7 ± 9.4	142.7 ± 8.0 *
Hematocrit (L/L)	0.43 ± 0.03	0.43 ± 0.04
MCV (fL)	53.0 ± 1.0	56.3 ± 1.8 ***
MCH (pg)	17.1 ± 0.8	18.3 ± 0.6 ***
MCHC (g/L)	320.6 ± 7.7	326.4 ± 6.0 *
Neutrophil-to-lymphocyte ratio (NLR)	0.61 ± 0.15	0.95 ± 0.28 ***
Systemic immune-inflammation index	464.0 ± 149.8	737.0 ± 363.9 **

^1^ Abbreviations: MCV—mean corpuscular volume; MCH—mean corpuscular hemoglobin; MCHC—mean corpuscular hemoglobin concentration. Results are presented as mean ± S.D. for 10 animals per strain. Statistically significant at: * *p* < 0.05, ** *p* < 0.01 and *** *p* < 0.001 for AO vs. DA rats.

**Table 3:** Fecal bacterial microbiota in healthy DA and AO rats ^1^.

	DA	AO
Alpha diversity		
Observed species	753.2 ± 165.9	807.5 ± 124.9
Shannon index	5.55 ± 1.36	5.87 ± 0.59
Chao1 index	847.9 ± 154.9	896.2 ± 170.1
Relative abundance		
Firmicutes	0.645 ± 0.168	0.708 ± 0.078
Bacteroidetes	0.179 ± 0.141	0.092 ± 0.064
Proteobacteria	0.054 ± 0.078	0.033 ± 0.020
Actinobacteria	0.070 ± 0.064	0.024 ± 0.010 **

^1^ Results are presented as mean ± S.D. for 10 animals per strain. Statistically significant at: ** *p* < 0.01 for AO vs. DA rats.

## Data Availability

The raw 16S rRNA gene sequence data reported in this study have been deposited in the European Nucleotide Archive (https://www.ebi.ac.uk/ena) under study accession number PRJEB60516. The other raw data supporting the conclusions of this article will be made available by the authors on request.

## Abbreviations

The following abbreviations are used in this manuscript:

DA rats	rats of Dark Agouti rat strain
AO rats	rats of Albino Oxford rat strain
HDL	high-density lipoprotein cholesterol
LDL	low-density lipoprotein cholesterol
AC	abdominal circumference
TC	thoracic circumference
BMI	body mass index
MCV	mean corpuscular volume
MCH	mean corpuscular hemoglobin
MCHC	mean corpuscular hemoglobin concentration
GTT	glucose tolerance test
ITT	insulin tolerance test
PT	prothrombin time
PTT	partial thromboplastin time
PDW	platelet distribution width
PCT	plateletcrit.
SOD	superoxide dismutase
CAT	catalase
MDA	malondialdehyde
PBMC	peripheral blood mononuclear cells
PMN	polymorphonuclear cells
IL	interleukin
IFN-γ	interferon gamma
TNF	tumor necrosis factor
MHC II	major histocompatibility complex class II molecule
LPS	lipopolysaccharide
TLR	toll-like receptors
